# Airway Epithelial Cells Condition Dendritic Cells to Express Multiple Immune Surveillance Genes

**DOI:** 10.1371/journal.pone.0044941

**Published:** 2012-09-11

**Authors:** Angela Rate, Anthony Bosco, Kathy L. McKenna, Patrick G. Holt, John W. Upham

**Affiliations:** 1 Telethon Institute for Child Health Research, and Centre for Child Health Research, The University of Western Australia, Perth, Western Australia, Australia; 2 School of Medicine, The University of Queensland, Brisbane, Queensland, Australia; Center of Ophtalmology, Germany

## Abstract

Increasing evidence suggests that crosstalk between airway epithelial cells (AEC) and adjacent dendritic cells (DC) tightly regulates airway mucosal DC function in steady state. AEC are known to express multiple immmuno-modulatory factors, though detailed information on how this influences human DC function remains incomplete. We recently demonstrated using an *in vitro* coculture model that AEC alter differentiation of monocytes into DC in a manner that inhibits expression of potentially damaging Th2 effector function. In the current study, we have extended these findings to examine other aspects of DC function. Using micro-array technology we show that multiple genes important for immune surveillance are significantly over expressed in purified AEC-conditioned DC, compared to control DC. These findings were confirmed by quantitative real time PCR or flow cytometry in an independent sample set. In particular, AEC-conditioned DC showed selective upregulation of chemokines that recruit Th1 cells, but minimal change in chemokines linked to Th2 cell recruitment. AEC-conditioned DC were also characterized by enhanced expression of complement family genes (*C1QB, C2, CD59* and *SERPING1*), Fcγ receptor genes (*FCGR1A*, *FCGR2A*, *FCGR2B* and *FCGR2C*), signaling lymphocytic activation molecule family member 1 (SLAM), programmed death ligands 1 and 2, CD54 and CD200R1, relative to control DC. These findings suggest that AEC conditioning facilitates the capacity of DC to react to danger signals, to enhance leukocyte recruitment, especially of Th1 effector cells, and to interact with other immune cell populations while minimizing the risks of excessive inflammation leading to tissue damage.

## Introduction

Respiratory tract dendritic cells (DC) are recognized as having a vital role in the regulation of immune responses to inhaled allergens, pollutants and pathogenic microbes [1]. DC are ubiquitous throughout the respiratory tract, forming a tight network of cells within the epithelium and submucosa of the conducting airways, the lung parenchyma and the nasal mucosa. These DC populations exhibit continuous turnover in steady state, a process that is accelerated in response to various inflammatory stimuli that induce the rapid migration into the lung of various DC precursor populations, including monocytes [2]–[5]. The intimate association of airway epithelial cells (AEC) and DC within the airway mucosa, and the plethora of mediators that both cell types can express, suggest that AEC are likely to play an important regulatory role in determining DC phenotype and function within the airways. Perturbations in these regulatory pathways are likely to be relevant to airway inflammatory disorders such as asthma. Though allergic sensitization and Th2 polarized immunity to inhaled allergens are important risk factors for asthma, only a proportion of allergic individuals develop asthma or atopic eczema, emphasizing the importance of specific regulatory factors within local tissue environments.

Several recent reports have shed light on the role of AEC in the regulation of DC function and the implications this has for both innate and adaptive immune function [6], [7]. Resting human AEC produce TGFβ at baseline that can selectively limit IL-12p70 and TNFα production by LPS-stimulated DC [8], suggesting that steady state AEC play a role in constraining the pro-inflammatory capacity of DC within the lung. Similarly, primary AEC from lung allografts can drive monocytes to differentiate into macrophages rather than DC [9]. Cytokine stimulated AEC can produce IL-15 that induces monocytes to differentiate into DC with some plasmacytoid features [10], while components of bacterial cells walls [11] and diesel exhaust particles [12], [13] can act via AEC to indirectly induce DC maturation. Importantly, it was recently shown in an experimental model of asthma that the ability of house dust mite allergen to induce DC activation and allergic inflammation was dependent on TLR4 expression on airway structural cells rather than on DC [14], thereby emphasizing that AEC regulate DC function, a role that is critical in the process of sensitization to inhaled allergens. Despite the fact that AEC have the potential to express an extensive range of immmuno-modulatory factors that can regulate the function of fully differentiated DC [6], [7], much less is known about the interactions between human AEC and monocytes during the initial phases of their differentiation into DC.

We recently reported a detailed analysis of AEC conditioning of DC using an *in vitro* model of cytokine-driven differentiation of monocytes into DC [15]. This model uses GM-CSF and IL-4 to drive the DC differentiation and is based on that used by Chomarat and co-workers to investigate stromal cell regulation of monocyte differentiation into either DC or macrophages [16]. By deliberately using purified CD14+ monocytes from allergen sensitized donors and by studying DC differentiation in the presence of GM-CSF and IL-4 (two cytokines that are enriched in airway mucosa of allergic asthmatics), we sought to study how AEC regulate DC function in a setting that is skewed toward the development of allergic inflammation.

After five days, AEC-conditioned monocyte derived DC (MDDC) were separated from AEC and purified by cell sorting prior to analysis [15]. Our results indicated that AEC modulate numerous aspects of DC phenotype and function in a contact dependent manner, effects that were observed with two AEC cell lines (16HBE and BEAS-2B). Using micro-array technology we then showed that over 1000 genes were differentially expressed (>2 fold change) in AEC conditioned MDDC versus control MDDC. Prominent among the differentially regulated genes in AEC conditioned MDDC were the type I interferon signaling pathway and the IL-6 signaling pathway. Blocking studies showed that type I IFN played a key role in AEC modulation of DC activation status, TLR3 and TLR4 signaling, and in the capacity of DC to induce Th1 and Th2 recall responses to allergens, while IL-6 modulated CD14 and CD40 expression on AEC-conditioned MDDC [15]. These findings led us to propose that steady state AEC modulate local DC differentiation within the airway mucosa, such that antimicrobial defenses are optimized, while simultaneously suppressing expression of Th2 immunity.

In addition, the microarray data highlighted significant changes in a variety of other genes that are relevant to DC function, especially the capacity of DC to react to danger signals and to interact with other immune cell populations. These gene families included chemokine genes, complement genes, Fcγ receptor genes and a variety of other immune response genes that were not examined in the previous publication [15]. The aim of the current study was therefore to validate these changes in gene expression in purified, AEC conditioned DC, using quantitative real time PCR analysis of RNA samples both from the original cells used for microarray, and in a separate set of experiments.

## Results

The type I interferon signaling pathway and the IL-6 signaling pathway were prominent among the genes showing higher expression in purified AEC-conditioned DC than in control DC, as detailed in our recent publication [15]. This was associated with prominent induction of type I interferons and IL-6 in AEC that were co-cultured with MDDC, as shown in Table 1. Blocking studies demonstrated that airway epithelial cell-derived type I interferon and IL-6 have distinct effects on DC phenotype and function.

**Table 1:** Expression of type I interferon and IL-6 in AEC co-cultured with MDDC.

	AEC	AEC(GM-CSF, IL-4)	AEC+MDDC(GM-CSF, IL-4)
IFNα2 mRNA	1.5±0.01	3.8±0.07 *	10.3±2.2 *
IFNβ mRNA	1.3±0.005	4.1±0.04 *	13.6±4.3 *
IL-6 (pg/ml)	<100	<100	26650±2200

AEC were cultured alone, in the presence or absence of GM-CSF + IL-4, or with MDDC. Following 5 days of culture RNA was extracted from sorted AEC and relative expression IFNα2 and IFNβ. *indicates p<0.05 relative to AEC alone (N = 6). IFNα protein was undetectable in culture supernatants. IL-6 protein data are from two experiments.

More detailed investigation of the microarray dataset with Ingenuity Pathway Analysis software (http:/www.ingenuity.com) highlighted that chemokine genes, complement family genes, Fcγ receptor genes and a variety of other immune response genes were also over expressed in AEC-conditioned MDDC than in control DC. These genes were undetectable in AEC cultured in the presence or absence of GM-CSF and IL-4 (data not shown). The following series of experiments sought to validate these findings using quantitative real-time PCR.

**Figure 1:**
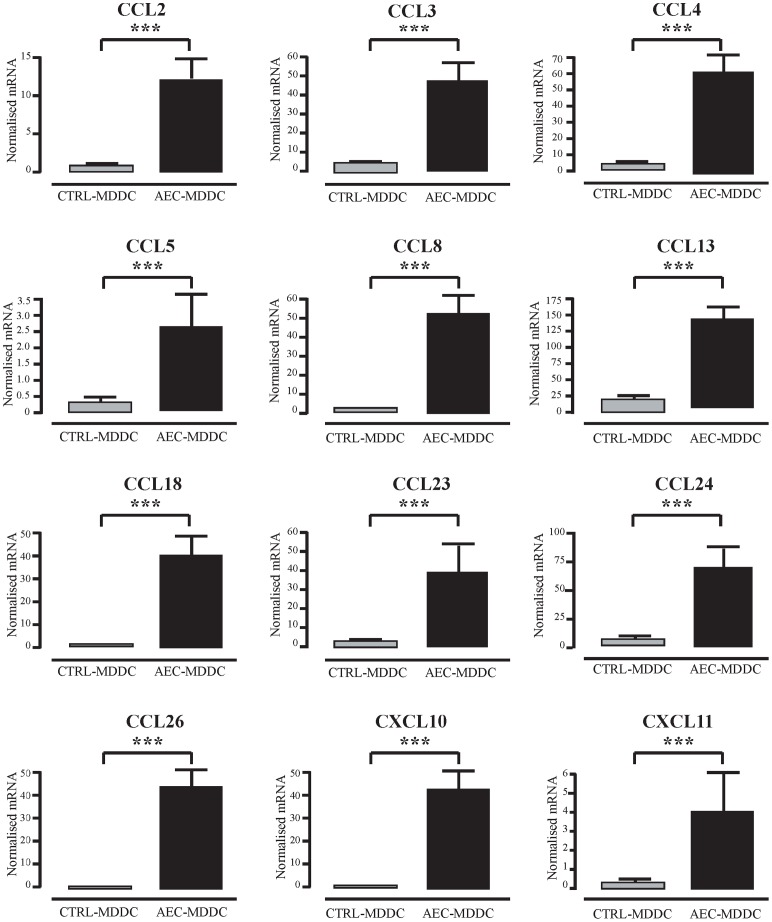
Airway epithelial cell-induced changes in DC expression of chemokine genes. After 5 days of culture in the presence or absence of AEC, DC were sorted by flow cytometry. RNA from 15 independent experiments was extracted, and expression of chemokine genes was determined using quantitative real-time PCR. *** p<0.001.

The microarray analysis identified twelve chemokines genes from the CC and CXC families of chemokines whose expression was upregulated in AEC-MDDC compared to the control-MDDC. These genes and their respective fold changes are outlined in Table 2. In order to validate over expression of chemokines, qRT-PCR analysis was first performed on RNA samples obtained from the initial 5 individuals, and then on an independent set of experiments with RNA from a further 10 individuals. Figure 1 shows the results of these PCR assays for all 15 paired samples. It is clear that differentiation of monocytes into DC in the presence of AEC resulted in MDDC that expressed significantly higher mRNA for all targets analyzed, compared to MDDC differentiated in the absence of AEC (p<0.001 for all). Thus, CCL2, CCL3, CCL4, CCL5, CCL8, CCL13, CCL18, CCL23, CCL24, CCL26, CXCL10 and CXCL11 were all expressed to a significantly greater extent in AEC-MDDC, than in control MDDC.

**Table 2:** Chemokine family members overexpressed by AEC-conditioned DC.

Chemokine	Alternative Name	Fold Change
**CCL2**	MCP-1	11.13
**CCL3**	MIP-1α	6.38
**CCL4**	MIP-1β	4.84
**CCL5**	RANTES	2.09
**CCL8**	MCP-2	66.01
**CCL13**	MCP-4	3.81
**CCL18**	PARC	19.33
**CCL23**	MPIF-1	5.82
**CCL24**	Eotaxin-2	7.60
**CCL26**	Eotaxin-3	5.36
**CXCL10**	IP-10	14.48
**CXCL11**	ITAC	6.68

Data was obtained by microarray analysis of five experiments. Fold change refers to the degree of over expression in AEC-conditioned DC compared with control DC.

**Figure 2:**
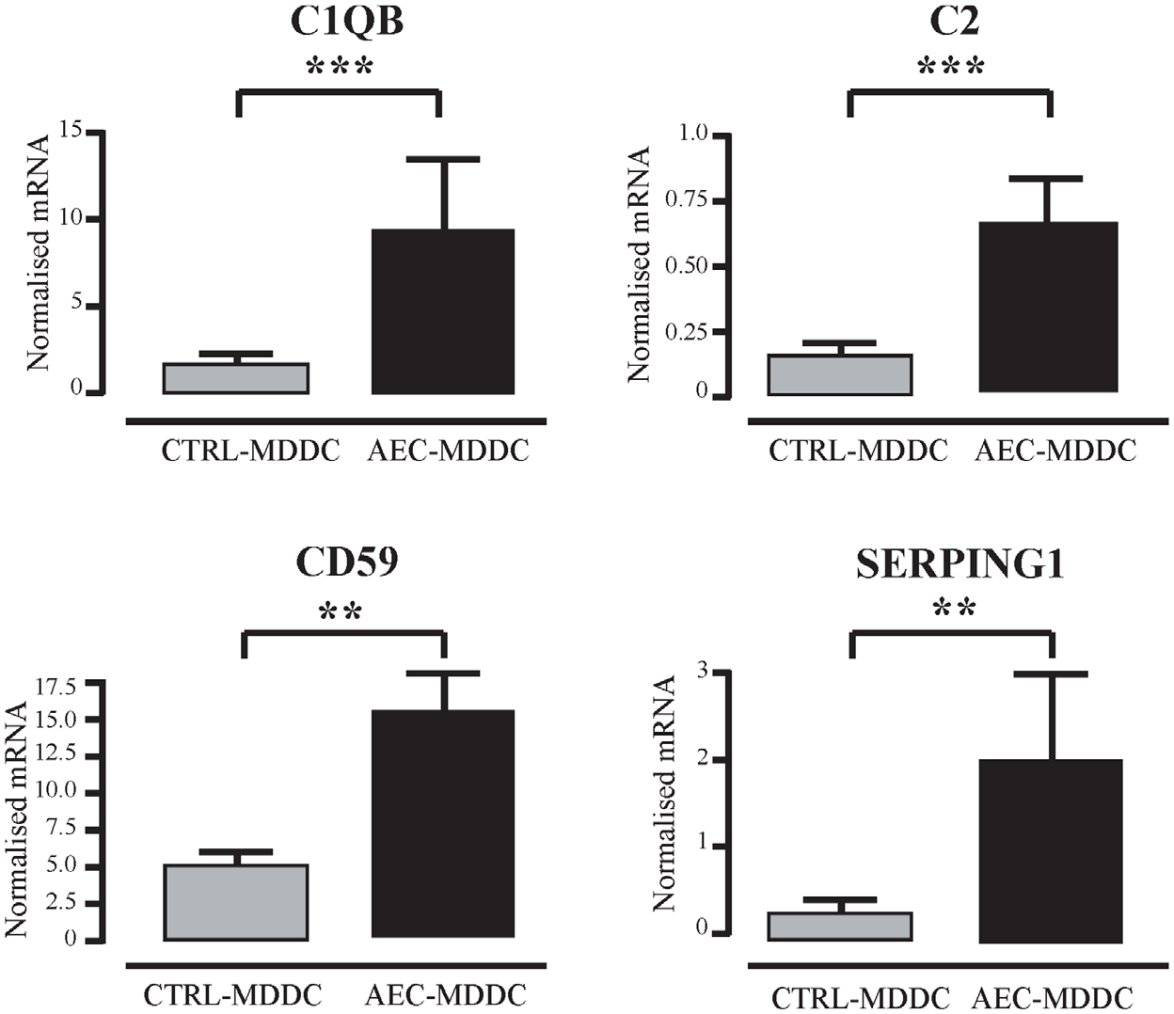
Airway epithelial cell-induced changes in DC expression of complement family genes. After 5 days of culture in the presence or absence of AEC, DC were sorted by flow cytometry. RNA from 15 independent experiments was extracted, and expression of complement pathway genes determined using quantitative real-time PCR. **p<0.01; ***p<0.001.

Human MDDC are known to produce not only complement components, but also complement inhibitors and their receptors [17]–[20]. The microarray analysis identified increased complement family gene expression in AEC-MDDC compared to the control-MDDC, including *C2*, *C1QB*, *C1R*, *C1S*, *C3AR1*, *CD59*, *CFB* and *SERPING1*. Of these eight genes, four were selected for further analysis by qRT-PCR on RNA samples from the 15 individuals. Differentiation of monocytes into DC in the presence of AEC resulted in MDDC that expressed significantly higher C1qb, C2, CD59 and SERPING1 mRNA, compared to control MDDC (Figure 2). Expression of the other four genes was not examined.

**Figure 3:**
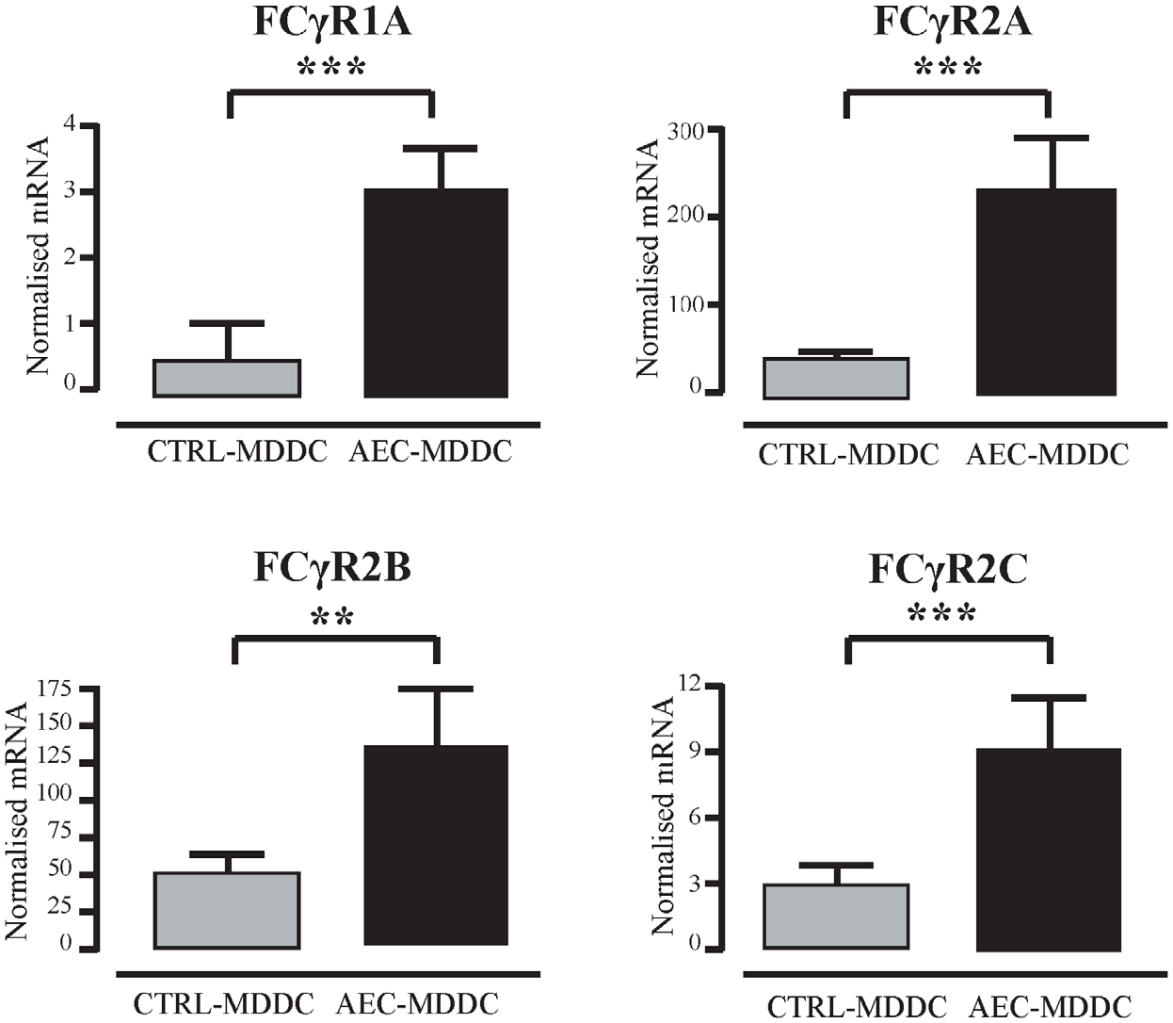
Airway epithelial cell-induced changes in DC expression of Fcγ receptor genes. After 5 days of culture in the presence or absence of AEC, DC were sorted by flow cytometry. RNA from 15 independent experiments was extracted, and expression of Fc gamma receptor genes was determined using quantitative real-time PCR. **p<0.01; ***p<0.001.

The Fcγ receptor family of genes encodes receptors for the Fc portion of IgG antibodies that are displayed on human DC [21]. The microarray analysis identified a number of Fcγ receptor genes, namely *FCGR1A*, *FCGR2A*, *FCGR2B*, *FCGR2C* and *FCGR3B*, so qRT-PCR analysis was performed in order to validate these findings. This confirmed that *FCGR1A*, *FCGR2A*, *FCGR2B* and *FCGR2C* mRNA transcripts were expressed to a significantly greater extent in AEC-MDDC compared to the control-MDDC, as detailed in Figure 3. In contrast, *FCGR3B* mRNA expression could not be detected in either MDDC subset by qRT-PCR in any experiments (data not shown).

**Figure 4:**
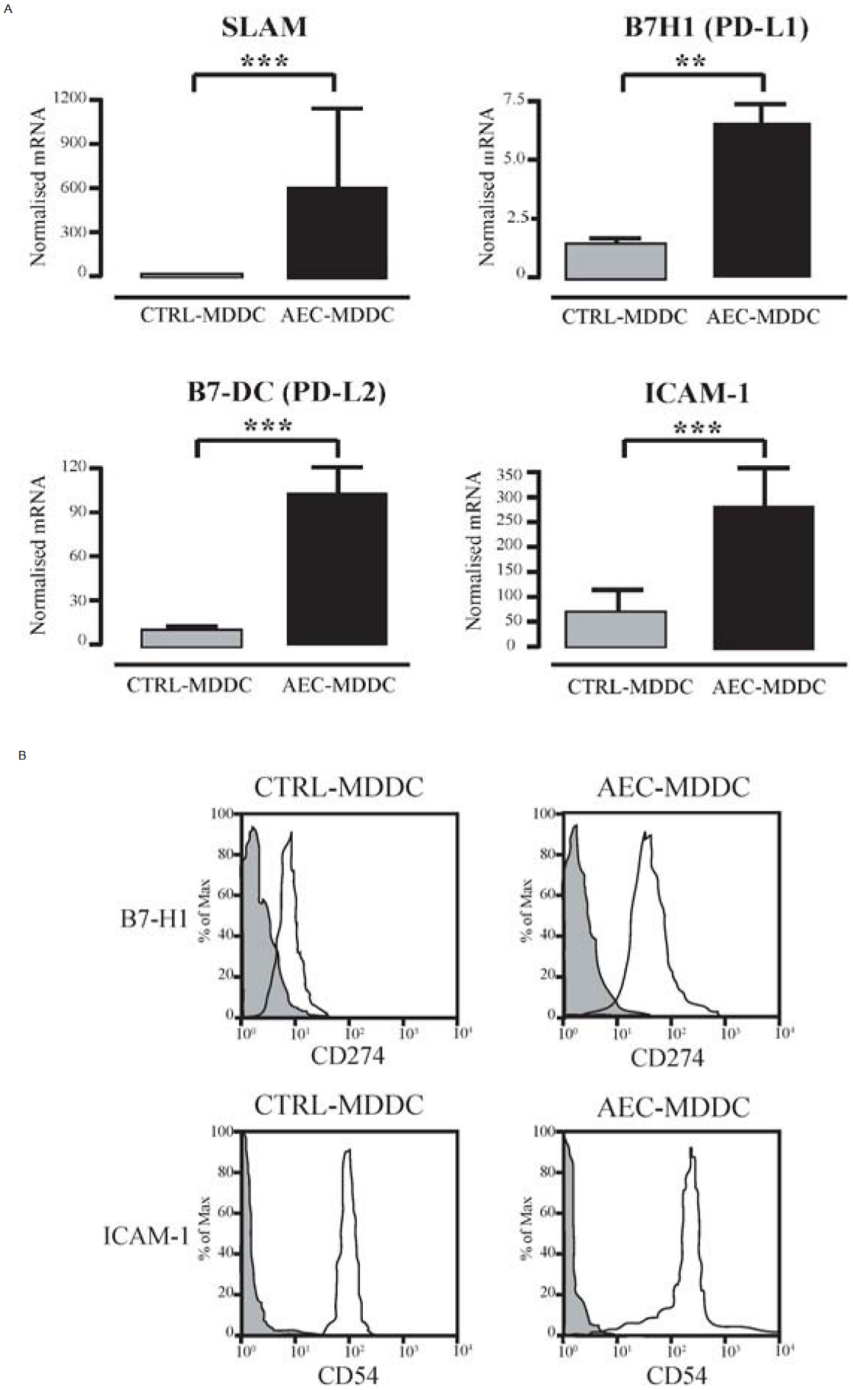
Airway epithelial cell-induced changes in DC expression of selected immune response genes. (A). After 5 days of culture in the presence or absence of AEC, DC were sorted by flow cytometry. RNA from 15 independent experiments was extracted, and expression of immune response genes was determined using quantitative real-time PCR. **p<0.01; ***p<0.001. (B) Cell surface expression of B7-H1 and ICAM-1 was determined by flow cytometry. Cells staining with specific antibody and isotype control antibodies are shown. Histograms from a representative experiment are shown. Similar changes were seen in all 8 experiments performed.

The microarray analysis also identified several immune response genes that are expressed on the surface of DC and which can modify DC function. qRT-PCR analysis of the 5 initial samples used in the microarray and 10 independent samples showed consistently higher mRNA expression of signaling lymphocytic activation molecule family member 1 (SLAM), programmed death ligand 1 (PD-L1, also known as CD274 or B7-H1), programmed death ligand 2 (PD-L2, also known as CD273 or B7-DC) and intercellular adhesion molecule 1 (ICAM-1 or CD54) in AEC-MDDC compared to control-MDDC (Figure 4A). For two of these markers, we confirmed increased protein expression of B7-H1 and ICAM-1 on the surface of the AEC-MDDC by flow cytometry (Figure 4B). B7-DC and SLAM were not examined by flow cytometry due to lack of available cell numbers.

**Figure 5:**
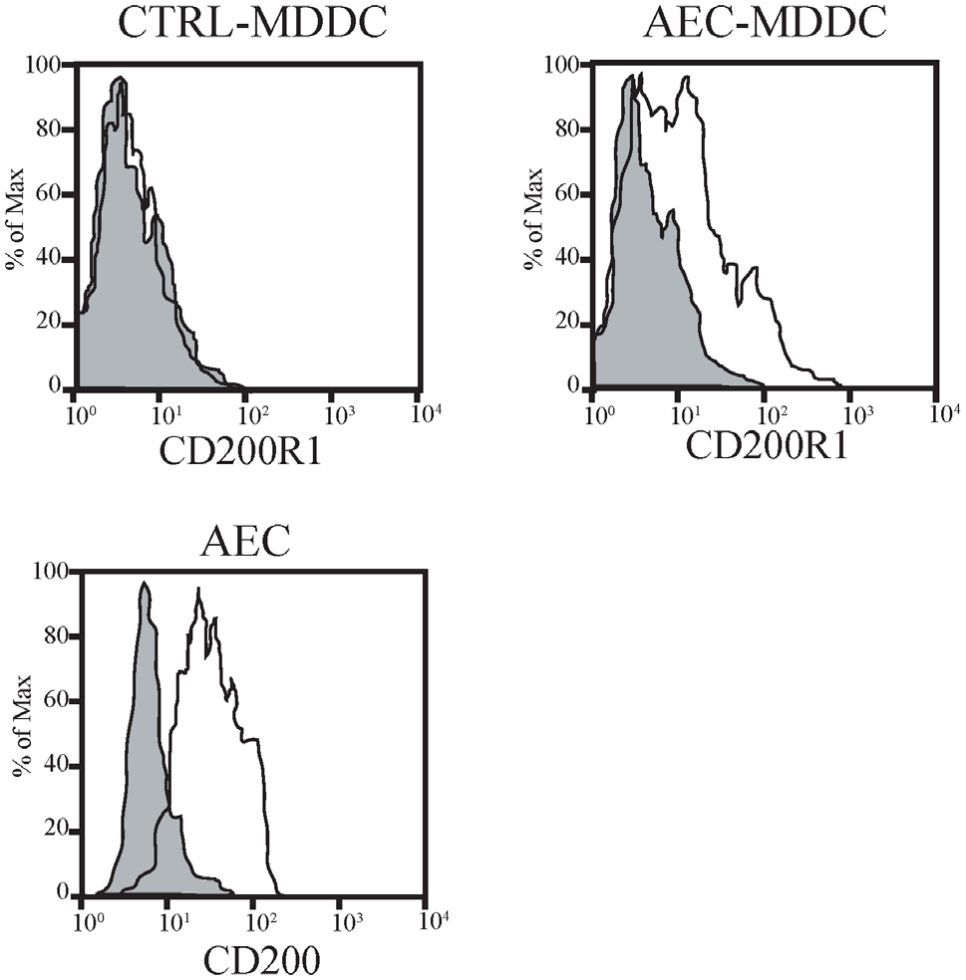
DC and airway epithelial cell expression of CD200R1 and CD200. After 5 days of culture in the presence or absence of AEC, cell surface expression of CD200R1 on DC and CD200 on AEC was determined by flow cytometry. Histograms from a representative experiment are shown. Similar changes were seen in all 6 experiments performed.

CD200R1 was identified in a list of genes determined to be statistically significantly higher in the AEC-MDDC subset using moderated *T*-test. qRT-PCR analysis confirmed higher CD200R1 mRNA expression in the AEC-MDDC cells compared to the ctrl-MDDC (p<0.001, n  = 15; data not shown). Flow cytometry showed negligible expression of CD200R1 on control MDDC, whereas moderate staining was identified on AEC-MDDC (Figure 5). Moreover, resting AEC expressed CD200 on their surface (Figure 5).

## Discussion

The key findings to emerge from the current study are that AEC conditioning of MDDC induces significant upregulation of a variety of genes and gene families that are likely to mediate important DC functions in the airway mucosa. These include genes with the capacity to direct recruitment of DC, their precursors and other immune effector cells (chemokines, complement), anti-microbial responses (complement, ICAM-1, SLAM), antigen uptake and processing (*FcGRs*) and interaction with T-cells (ICAM-1, B7-H1, B7-DC, SLAM). In most instances we were able to verify the initial microarray findings using a different technique (quantitative real-time PCR or flow cytometry) in the same subjects, and also in an additional independent group of experiments from ten individuals.

DC can express a distinct pattern of chemokines, both constitutively and upon activation with inflammatory stimuli [22]. Based on the finding described herein, it appears that AEC may participate in the regulation of chemokine production by local DC populations during their differentiation within the airway mucosa. A unique attribute of airway mucosal DC networks is their extremely high turnover rate in the steady state due to the continuous sampling of the local antigenic environment [23]. Many of the chemokines expressed in the AEC-conditioned MDDC play an active role in the recruitment of immature DC and monocytes and may thus participate as critical players in driving their own replacement. It is noteworthy that at baseline, the recruitment of DC into the lung is highly dependent on CCR1 and CCR5 expression [24], and that six out of the twelve upregulated chemokines shown in Figure 1 function as agonists for CCR1 and/or CCR5. This high baseline expression of a number of chemokine family members in the AEC-conditioned MDDC subset is in keeping with a recent study of murine lung DC subsets that observed that CD11b+ DC express 16 different chemokine mRNA transcripts at baseline, including CCL3, CCL4, CCL5 and CXCL10 [22].

Notably, there appeared to be a preferential bias towards the selective upregulation of chemokines that recruit Th1 cells such as CCL3, CCL4, CCL5, CXCL10 and CXCL11 [25], [26]. In contrast, chemokines such as CCL1, CCL17, CCL21 and CCL22 which have been previously linked to chemotaxis of Th2 effector T-cells [27], [28] were not differentially expressed between AEC-conditioned and control MDDC. Exposure of monocytes to type I IFNs during their differentiation to DC selectively upregulates Th1-associated chemokines including CXCL10 [29], and given our recent findings of enhanced type I IFN signaling in AEC-conditioned MDDC, this provides a plausible mechanism by which such DC might attain this specific chemokine profile. The finding of increased expression of Th1 attracting chemokines by AEC-conditioned DC complements our recent observation that AEC-conditioned DC selectively attenuate allergen-specific Th2 cytokine synthesis, while leaving Th1 responses intact [15].

The complement system provides a crucial component of anti-microbial host defense in the airways, though excessive complement activations can lead to immunopathology. There is good evidence that DC can express complement proteins both at baseline and after stimulation [18]. A number of factors that inhibit complement function were enriched in the AEC-MDDC transcriptome. CD59, also known as ‘protectin’, is a surface-expressed molecule that is present on host cells and prevents the formation of the lytic membrane attack complex, and in this context may operate to protect the host DC from the effects of locally-produced complement following exposure to inhaled pathogens [30]. Mice that are genetically deficient in CD59 show increased lung immunopathology following influenza infection [31]. SERPING1, a serine protease inhibitor, encodes a highly glycosylated protein that has been shown to have inhibitory effects on complement activation pathways [32]. Furthermore, expression of C1qb by DC can bind to apoptotic cells and facilitate their clearance, thus contributing to the overall resolution of an immune response following infection [33]. Thus, whilst AEC-conditioned DC may contribute to the local activation of complement pathways, they may also be equipped to ensure against collateral damage to host cells induced by these effector molecules.

AEC-conditioned DC showed enhanced expression FCGR1A, FCGR2A, FCGR2C, and FCGR2B, compared with control DC. This would be predicted to facilitate antigen sampling by DC, and in particular may promote cross-presentation of exogenous antigens in the context of pulmonary viral infection [34]. Human lung DC express FcγRI [35], though it is unclear whether they express FcγRII or FcγRIII molecules. As well as their role in internalizing exogenous antigen, ligation of surface FcγRs DC can modulate DC function by triggering of intracellular motifs that can activate or inhibit cell function [21]. FcγRIa, FcγRIIa and FcγRIIc are all linked to cellular activation, whereas FcγRIIb is associated instead with reduced DC phagocytosis and TNF-a production [36] and more broadly to tolerance to innocuous antigens [37]. There is precedent in the literature for co-expression of multiple Fcγ receptors on the DC [21], [36] and it may be predicted that the balance of activating and inhibitory Fcγ receptors on DC could function as a control point for regulation of DC within the airway epithelium.

SLAM (also known as CD150 and SLAMF1) is a self-ligand receptor present on the surface of activated DC and a receptor for measles virus. Engagement of SLAM has been shown to skew allergic Th2 effector cells towards a Th1 phenotype [38]. ICAM-1 is an adhesion molecule and the main receptor for the major subtypes of human rhinoviruses. It is highly expressed on human lung DC [39], and similar to SLAM, it appears that ICAM-1/LFA-1 interaction promotes Th1 immunity during initial polarization of naïve T-cells [40]. The capacity of AEC to augment SLAM and ICAM-1 expression on DC (Figure 4A and 4B), together with enhanced TLR3 and type I IFN expression [15], is likely to be important for optimizing host defence against a variety of respiratory viruses. PD-L1 and PD-L2 are both highly expressed on airway DC within the epithelium [41], and are thought to regulate T-cell activation and tolerance. These two molecules have important but distinct effects in experimental models of allergic airway inflammation [42], [43].

Emerging evidence points to a role for the negative regulatory molecule CD200 and its receptor CD200R in the maintenance of immunological homeostasis within the lungs [44]. CD200R1 is expressed on alveolar macrophages and lung DC while CD200 is expressed on epithelial cells, and mice deficient in CD200 show increased mortality and delayed resolution of airway inflammation following influenza infection [45].

In our earlier publication we examined how AEC modulate the differentiation of monocytes into DC ^15^. As shown in Table 1, co-culture of AEC with MDDC induces AEC to express type I interferons and IL-6. In the earlier publication we confirmed the biological importance of these cytokines using blocking strategies to show that airway epithelial cell-derived type I interferon and IL-6 have distinct effects on DC phenotype and function ^15^. The current study extends these observations to show by gene microarray that AEC also modify the expression of multiple other genes. The findings were confirmed in two ways – verification by quantitative real time PCR in an independent set of experiments, and in some instances we were able to demonstrate that these changes in mRNA expression were accompanied by changes in protein expression by e.g. B7-H1, ICAM-1 and CD200/CD200R1 (see Figures 4 and 5). In other instances, lack of sufficient cells and/or culture supernatant precluded further protein measurements.

Our experiments used a bronchial epithelial cell line, and monocyte derived DC, so it will important for future studies to verify our findings using primary AEC cultures co-cultured with DC precursors isolated directly from peripheral blood. Experiment with human pre-DC precursors would be of interest, but because such cells are so infrequent within the peripheral blood, this would restrict the range of experiments that could be performed. It will also be important for future studies to compare the findings with DC isolated directly from the airway mucosa of healthy individuals. Our findings also have important implications for inflammatory diseases such as asthma where epithelial cell dysfunction and DC activation are prominent features: detailed studies of AEC:DC interactions in asthma should be an important priority.

In summary, the results presented herein provide additional detailed information on the influence of AEC on the differentiation of DC from monocyte precursors. AEC conditioning facilitates a number of DC functions important in the airway mucosa including direct host defence against pathogenic microbes (complement, ICAM-1, SLAM), recruitment of DC, their precursors and other immune effector cells (chemokines, complement), antigen uptake and processing (FcγRs) and interaction with T-cells (ICAM-1, B7-H1, B7-DC, SLAM). Many of these molecules can modulate DC function directly and enhance the responsiveness of DC to mediators within the airway mucosal environment, while at the same time keeping steady state DC relatively unresponsive to inhaled innocuous antigens.

## Materials and Methods

### Cell Separation and Co-culture

The study was conducted in accordance to the Declaration of Helsinki and was approved by the Ethics Committee, Princess Margaret Hospital, Perth. Written informed consent was obtained from each study subject. After obtaining informed consent, blood samples were obtained from healthy adult volunteers (age 21–65 years) all of whom were sensitized to house dust mite (*Dermatophagoides pteronyssinus*) with a mean wheal diameter of 3 mm or greater on allergy skin testing. None of the subjects had symptoms of allergic diseases such as asthma or eczema. Blood monocyte isolation was performed as described previously [15]. Briefly, CD14+ monocytes were isolated from peripheral blood mononuclear cells using MACS beads (Miltenyi Biotech, Germany) according to the manufacturer’s directions and resuspended in complete media containing 5% fetal calf serum, recombinant IL-4 and GM-CSF.

Mycoplasma-free 16HBE 14o- cells were used as previously described [15]. These cells exhibit well defined tight junctions and gap junctions and are morphologically similar to basal AECs *in vivo*. 16HBE cells and are thus regarded as a reasonable surrogate for the basal AECs that are in direct contact with intraepithelial DC *in vivo*. These 16HBE were seeded in 24-well culture plates at a density of 10^4^ cells per cm^2^ in order to achieve approximately 70% confluence prior to addition of monocytes.

Monocytes were cultured alone or added to semi-confluent 16HBE cells such that the monocytes were able to take up position between AEC. After 5 days, viable CD11c+ MDDC were separated from AEC and sorted using a FACSAria flow cytometer (Becton Dickinson, USA) as described in detail elsewhere [15]. The purity of CD11c+ sorted cells that were negative for propidium iodide was routinely above 97%. In some experiments, the absence of contaminating AEC was confirmed by staining for intracellular cytokeratin using a FITC-conjugated anti-cytokeratin antibody (BD Biosciences, USA).

The surface phenotype of MDDC was determined [15], using antibodies against CD54/ICAM-1 and CD274/B7-H1 (both from eBiosciences, San Diego, CA), CD200 and CD200R1 (both from R&D Systems, Minneapolis, MN) and appropriate isotype controls. Cells were washed, fixed and analyzed within 24 hours on a FACSCalibur flow cytometer (BD Pharmingen, USA) using CellQuest and FlowJo software.

### Microarray and Quantitative Real-time PCR

Total RNA isolation, reverse transcription and target mRNA quantification by real-time PCR were performed [15]. Copy numbers were determined in 10-fold serial dilutions of plasmid standards and normalized to the reference gene *EEF1A1*. Total RNA samples from 5 donors were labeled and hybridized to U133 Plus 2.0 microarrays (Affymetrix) as previously described [15]. Details of the microarray data can be viewed at http://www.ncbi.nlm.nih.gov/geo (Gene Expression Omnibus accession number GSE12773).

Microarray data were analyzed in the *R* environment for statistical computing (www.r-project.org/) as detailed in Methods S1. Differentially expressed genes were identified using moderated *t* statistics [46] with false discovery rate control for multiple hypothesis testing [47]. Differentially expressed genes were ranked based on their fold change values and screened for membership of known biological pathways using specialized software as detailed in Methods S1. Selected over expressed genes were validated by quantitative real-time PCR or flow cytometry in an independent sample set (n = 10 additional donors) according to the methodology outlined above.

### Statistical Analysis

Group data are expressed as mean ± SE. The significance of differences between paired samples was compared by Wilcoxon signed-rank test using SPSS version 11 for Macintosh. P<0.05 was considered statistically significant.

## Supporting Information

Methods S1 (DOC)
