# Supplementary material for: Airway Epithelial Cells Condition Dendritic Cells to Express Multiple Immune Surveillance Genes
Source: PLoS One. 2012 Sep 11;7(9):e44941. doi: 10.1371/journal.pone.0044941 (PMC3439377; doi:10.1371/journal.pone.0044941)
Supplement: Methods S1 — (DOC) [file pone.0044941.s001.doc]

# Supplementary Methods

Microarray data were analyzed in the *R* environment for statistical computing (www.r-project.org/) using the affyPLM (probe level model) algorithm for data preprocessing [48]. Differentially expressed genes were identified using moderated *t* statistics [46] with false discovery rate control for multiple hypothesis testing [47]. The differentially expressed genes (false discovery rate cutoff, <0.01) were ranked based on their fold change values and were screened for membership of known biological pathways. The Gene Expression Omnibus accession number is GSE12773; details of the data can be viewed at http://www.ncbi.nlm.nih.gov/geo.

Specialized software was employed to identify biological pathways that were significantly enriched. Differentially expressed genes were uploaded into the Ingenuity Pathway Analysis software (http://www.ingenuity.com), in order to dynamically generate significant regulatory and signaling networks or pathways. Canonical pathway analysis used annotated pathway maps to project the genes from a given pathway that is shown to be active. The significance of a canonical pathway is controlled by *p*-value, which is calculated using the right-tailed (referring to the overrepresented pathway) Fisher Exact Test for 2x2 contingency tables. This is done by comparing the number of ‘Focus’ genes that participate in a given pathway, relative to the total number of occurrences of those genes in all pathways stored in the IPKB. The significance threshold of a canonical pathway is set to 1.3, which is derived by –log10 [*p*-value], with *p* value ≤ 0.05. Selected over expressed genes were validated by quantitative real-time PCR in an independent sample set (n=10) according to the methodology outlined above.
